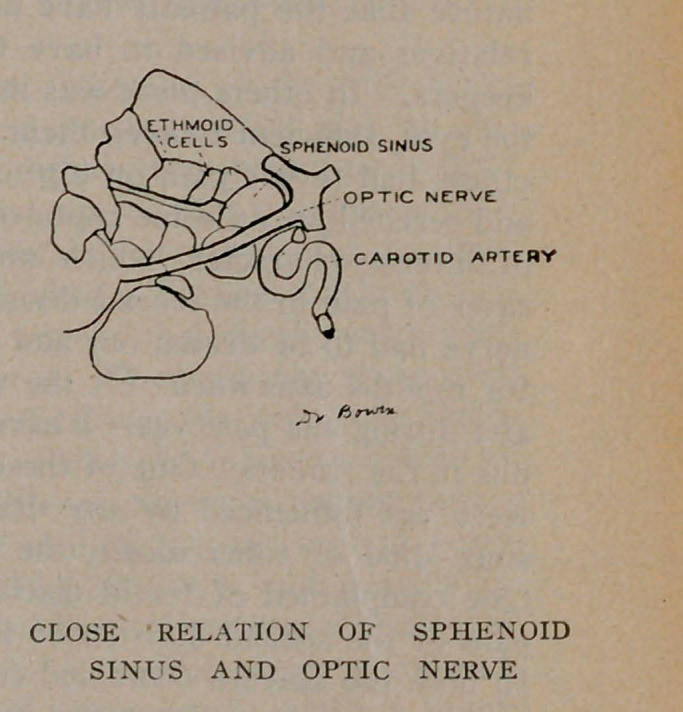# Latent Sinusitus

**Published:** 1911-08

**Authors:** George F. Cott

**Affiliations:** Buffalo, N. Y.; 1195 Main Street


### BUFFALO MEDICAL JOURNAL

VOL. LXVII. AUGUST, 1911. No. 1

### ORIGINAL COMMUNICATIONS

### Latent Sinusitus

By GEORGE F. COTT, M.D.
Buffalo, N. Y.

INDEX
THROUGH

THE four sinuses of the skull, frontal sinus, ethmoid cells, sphenoid sinus and antrum of Highmore, have been made the subject of many papers. The antrum of Highmore, however, held the field against all comers and was referred to the dentist mostly, as properly belonging to his field for treatment; and perhaps justly so, since so often the antrum became infected through the root of a tooth. About twenty years ago, however, during the epidemic of American grippe so aptly described by Carl Seiler in Burnett's Otology, conditions changed. The other sinuses became more involved and were left crippled to give further trouble years later. The immediate effect of the epidemic was to leave the cell cavities denuded and the nerves exposed lying upon the bone unprotected by their periosteal covering which left them in a condition readily to be acted upon by some peculiar irritant in the air. The apparent causative factor is a mixed infection not due to the influenza or grippe germ. In our city, as observed by me since the first epidemic, the antrum of Highmore became the offending organ a number of years after the epidemic. Pus was invariably found upon opening that cavity, always denuded of its membrane, and at times foul granulations and quantities of polypoid degenerations were removed, with uniformly good results. After the antrum disease had run its course the ethmoid cells became troublesome and for several years they received apparently exclusive attention. Then that subsided and the frontal sinus came into play. The sphenoid then showed considerable unrest and was heard from next with all its dire consequences. During 1910 the maxillary sinus again became very prominent in surgery. In other parts of the country the rotation was not so exact as I have noticed it, the different sinuses receiving attention from time to time and had to be radically treated.

Disease of the sinuses is far more common in the adult than in children because , first the sinuses are not so fully developed in the young, and secondly they seem to be more or less immune as compared to the adult; per contra, the child suffers much more frequently from involvement of the middle ear from disease of the throat thru the Eustachian tube than does the adult.

The sinuses are located as follows: Frontal sinus in the frontal bone, its inner wall forming part of the outer wall of the cranial cavity. It communicates with the brain by blood vessels perforating the inner plate.

RIGHT OPTIC NERVE MORE EXPOSED THAN LEFT

The ethmoid cells are located immediately under the frontal lobes in the median line, the separating cortex being very thin and perforated by numerous foramina for the olfactory nerve. The anterior and posterior ethmoid arteries and nerves also penetrate the bone.

The sphenoid sinuses are located in the body of the sphenoid bone separated from each other by a thin bony septum. They are very irregular in shape; the one often differs from its fellow of the opposite side. All these sinuses occur in pairs. The optic nerve runs close by the sphenoid sinus and sometimes encroaches upon its cavity, as will be noticed from the plates.

The antrum of Highmore lies in the superior maxillary bone and its outer wall forms the prominence of the cheek; its inner wall—thin plates of bone—forms part of the outer wall of the nasal cavity and its roof is composed of the orbital plate. The second branch of the fifth nerve runs along in a groove under the orbit and occasionally penetrates its plate.

These different sinuses are covered with periosteum and mucous membrane. They all open into the nose, as follows: the frontal sinus through the infundibulum opens into the middle meatus; the anterior ethmoid cells and the the antrum of Highmore likewise into the middle meatus.

OPTIC NERVE AT MARGIN OF POSTERIOR ETHMOID CELL

The posterior ethmoid cells open into the superior meatus with the sphenoid sinus. Thus it will be seen that infection can readily enter any or all of these air spaces thru their natural openings.

These spaces are natural cavities in the bones of the skull and are developed as follows: The frontal sinus is absent at birth, develops about the 7th year of life and gradually increases until the age of 20. They are often unequal in size and vary in extent in different individuals. Some are very small on one or both sides and sometimes extend away up into the frontal bone. The cavities may contain trabeculae of irregular size.

The sinuses are separated by a septum which may contain an opening. Occasionally they communicate with a cell in the Crista Galli. In a few cases it has been noticed that the sinuses are not developed at all.

The ethmoid cells are not developed until the fourth year, excepting perhaps the rudimentary bulla and another small space. The outer surface of the lateral mass of the ethmoid is formed by a thin plate of bone called the os planum or lamina papyracia and forms the inner wall of the orbit. In some cases this plate of bone never develops and the sinuses are separated from the orbit by membrane only.

BOTH NERVES OUT OF DANGER ZONE

ONE NERVE IN DANGER OF INFECTION

The sphenoid sinuses do not exist in early childhood but develop after the seventh year, or from that to the twentieth. Occasionally one sinus or both may be absent. In his investigation Onodi has found thirty-eight varieties of sphenoid cells, that is in shape, size, location and difference between the two cavities. One may be very much larger than the other. They increase in size as age advances. They have been found to extend in the pterygoid process and base of the greater wing of the sphenoid bone or extend into the basilar process of the occipital bone nearly to the formen magnum (Gray).

The antrum of Highmore is rudimentary at birth and gradually increases to its full size at puberty.

I have gone into some detail regarding the anatomy of these sinuses because operations are so frequently performed upon them by surgeons and near-surgeons. If the peculiar and varied shapes and sizes of the different cavities are not borne in mind some avoidable accidents with most serious consequences are apt to follow.

To come back now to the diseases which commonly affect the sinuses. The antrum of Highmore was mostly infected from carious roots of teeth, occasionally by zymotic disease so commonly affecting the throat and nose; but since the advent of grippe or influenza all other causes have been put into the shade and the millions of sinus cases now extant are by a large majority due to its destructive influence. Those of us who practised during the epidemic twenty years ago know how serious were the cases we encountered daily; pain in back and limbs, nasal rhinorrhoea, after some days formation of pus in profuse quantities in the nose, intense headache to the verge of delirium.

OPTIC CANAL RUNS THROUGH SINUS

CLOSE RELATION OF SPHENOID SINUS AND OPTIC NERVE

Since we know more of the effects produced by this virulent poison we can readily explain the source of pain. The dura mater is supplied with nerve filaments from the sympathetic, the 4th, 5th and 12th nerves while the pia mater is supplied by filaments of the 3rd, 5, 6, 7, 9, 10 and 11th cranial nerves, some of these as you will observe are motor nerves, but each one receives sensory filaments from the tri-geminus, glosso-pharyngeal or pneumo-gastric. The effect of pain is not due to brain involvement but to neuritis which may extend throughout the sensory nerve influence, though brain abscess or meningitis has occurred principally when pus was found in the sinuses. In the present state of sinus disease however, we do not always find pus, the sinuses usually being dry, but the nerves exposed and through some peculiar external irritation the mucus membrane of the nose becomes swollen, drainage becomes defective, or, where this is not the case, the peculiar irritation acts upon the exposed nerves without the nasal mucous membrane having become involved primarily and causes neuritis with all its painful and destructive influences.

In the first epidemic, pneumonia was a great source of revenue to the undertaker. Most all patients who had a severe attack of grippe at that time or since and escaped pneumonia and apparently recovered have had some defect which lowered their resisting power. One of these is denudation of the sinuses and expôsure of the nerves either one or more, and these lie quiescent until some sort of semi- or quassi-epidemic calls upon this sensory nerve supply to help along the inglorious work of pain. One of the principal symptoms is pain and this is of so intense a nature that the patients have actually been adjudged insane by relatives and advised to have them removed to the custody of keepers. In others there was intense pressure immediately above the eyes, sufficient to keep them from their usual occupation; still others had severe pain on top of the head and others at the base and cerebellum; in some supra-orbital; in others infra-orbital pain of intense character, which nothing relieved. I know of two cases of pain in the second division of the 5th in which finally the nerve had to be drawn out and then the patients did not recover for months afterward. Of the numerous patients suffering now, and during the past year, I have met but three who actually had pus in the sinuses. One of these had pus in all the sinuses which were not influenced by any drainage or washings out, and has since died of tuberculosis, the other two were antrum disease. One complained of foetid discharge, the other of discharge and pain of the second division of the fifth and supra-orbital region. In both the antrum contained foul smelling granulations.

These patients also suffer mentally not to a point of insanity but sufficiently to keep them from attending to business or work. At times during the wet, damp or dusty season they complain of mouth breathing, or of easily catching cold, winding up with purulent discharge. These so-called colds are not easily amenable to treatment but seem to extend over several weeks or months before they can be made to yield. In addition to the swelling of the mucous membrane of the nasal cavities we find the throat affected; this however is usually cursorily only, but the trachea suffers most, and occasionally the bronchi. Recently some feebly resistant patients were furthermore afflicted by developing pleuropneumonia with an accumulation of fluid in the pleural cavity which when withdrawn consisted of blood instead of serum or pus (Buswell). What other phase this terrible scourge will next exhibit we must anxiously await. I certainly believe that in the last twenty years more people died of grippe, or its consequences than many destructive diseases put together, the certificate of death giving the cause as some particular ailment, but fundamentally due to latent grippe.

The principal objective symptom affecting the trachea is intense congestion down to and beyond the bifurcation. The mucous membrane of the pharynx and larynx is generally involved. The subjective symptom is cough, which may occur every few minutes and often spasmodically during the night keeping the patient from sleep. This cough consists more of a bark than anything else, causing pain in the lower chest due mostly to muscular strain and to sensitive nerve areas at the bifurcation, spasm of the glottis, headache and fatigue due to want of rest. Exceptionally is anything raised by the tremendous exertions. This tracheitis often lasts a month or two but finally improves with or without treatment.

The pain can often be traced to branches of the fifth nerve although the glosso-pharyngeal and pneumogastric play an important part in its production. It differs from neuralgia by first being traced to a specific cause and not of that peculiar periodically intense nature affecting a certain nerve but of a constant depressing or pressure-like pain, unless a separate branch is affected when the pain will be so intense that death seems preferable to these patients than to bear it. The pain may show itself in other parts of the nerve not periodically as we commonly find in neuralgia but lasting indefinitely, of varying intensity not at all uniform in character, but in its different varieties lasting over a long period, recurring milder and milder, say for several years. A very important difference between neuralgia and neuritis involving the cranial nerves is the effect of treatment. Neuralgia is obstreperous and obstinate, while neuritis yields quite readily, in many cases immediately and permanently while in others cessation of pain is apparently a remote effect of treatment while in a third class all symptoms vanish to re-appear, mildly or severely for a few weeks then stay away; a few remain severe whenever an attack occurs.

Thus, has been this peculiar cycle of the latent effects of grippe. The antrum of Highmore suffered mostly for several years and was usually only relieved by operation. Following upon this we had a period of ethmoid disease also relieved by operation; then the frontal sinus caused more trouble, and the others seem to have been forgotten, excepting the sphenoid, which lately sprang into prominence and now again the antrum of Highmore. I do not mean that these different sinuses are not in a receptive state but irritation seems by predilection to call forth symptoms referable to a particular sinus only, or the nerves connected with that sinus.

For purposes of differentiation let us divide the sinus affections into suppurative, hyperplastic and dry stages, the latter being the latent stage or that one following suppuration. The suppurative and hyperplastic stages may and do occur independently while the dry stage always follows suppuration.

The latent stage seems harmless unless called into activity by some irritant. Such patients are susceptible to rapid changes of temperature, to dust and to high humidity. They complain of colds which do not seem to get well. The secretion when present in such cases is viscid and commonly clings to the pharyngeal wall and occasionally the effort to remove it causes vomiting; when abundant it escapes down the oesophagus at night causing indigestion and diarrhea and consequent emaciation in instances to a point of starvation. In another class of cases in this stage the mucous membrane and periosteum have been destroyed and the nerves exposed. Now when drainage has been obstructed, either pneumatic or exudative, there is always a chance for trouble. This may come during subacute attacks or periodical colds or without any apparent cause. The commonest remote effect is neuritis involving principally the fifth nerve. Now if we follow up the distribution of this sensory nerve we readily find an answer for the causation of the severe pain. I do not lay much stress upon the so-called localized meningitis in those cases of long standing but consider them nearly always as neuritis. Compare their actions with nerves inflamed anywhere else in the body; the result is practically the same. Very many patients recover and a few withstand their sufferings for years.

A very important effect of latent sinusitis is blindness. The sphenoid sinus and the optic nerve are very close associates, and may be in actual contact by dehiscence, or weakening of the optic canal where it forms a part of the sinus wall and thereby the nerve easily becomes infected producing optic neuritis and consequently blindness, as I would apply the term deafness, being of all degrees. We may have only certain areas affected which is designated as scotoma, or the entire fundus may have become involved. None of the dozen or so cases which I saw lately were totally blind because the infection is very much milder than that causing pus formation. This blindness seems to come on quite suddenly, perhaps over night and gradually seems to grow worse, usually accompanied by a sense of pressure over the eyes or on top of the head. In a few patients no subjective symptoms were present. The symptoms seem so sudden that fortunately the patient seeks to ascertain the cause of the defect of vision. Nor is the faulty vision very mild; some cannot distinguish objects across the room while others cannot make them out a foot from the eyes. Where certain fields of the nerve are affected moving objects can only be seen at a certain angle or in an irregular circle in extreme divergence or near the median line then again vanish. Happily the present quassi-epidemic is within our power and patients can usually be relieved by operation; some however have been suffering for several years and were quite or totally incapacitated from work of any kind. Their trouble was not diagnosticated for a long time, but after once located and treated gradually recovered.

1195 MAIN STREET.

## Figures and Tables

**Figure f1:**
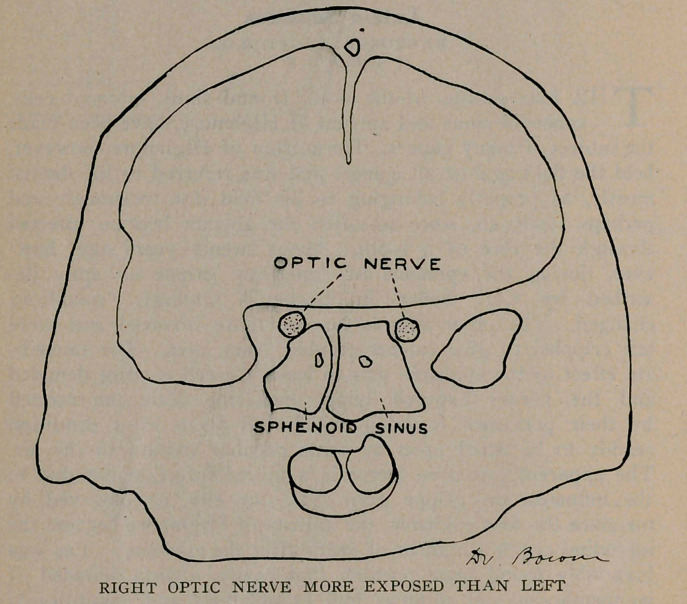


**Figure f2:**
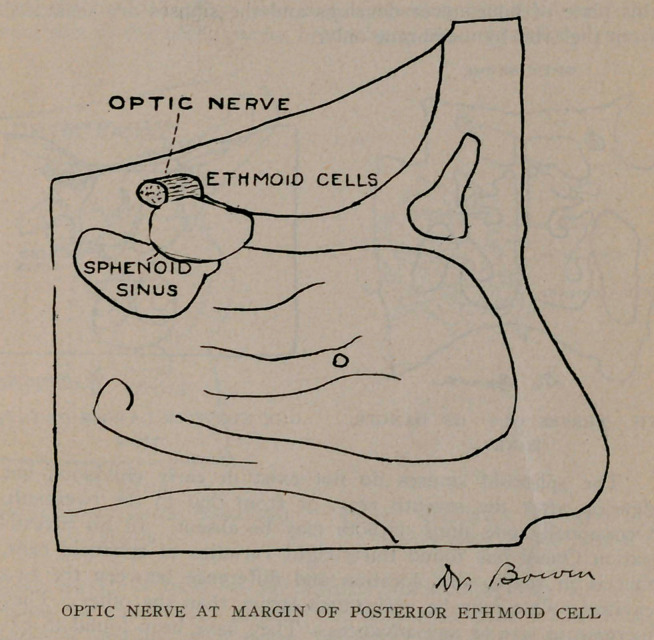


**Figure f3:**
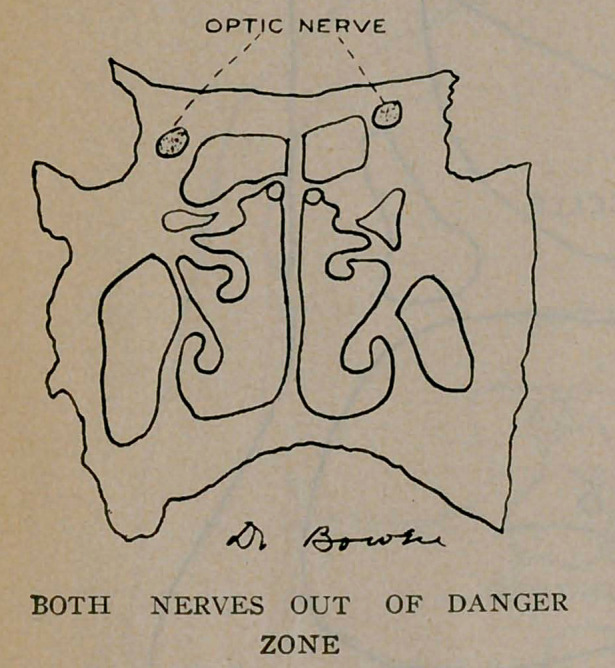


**Figure f4:**
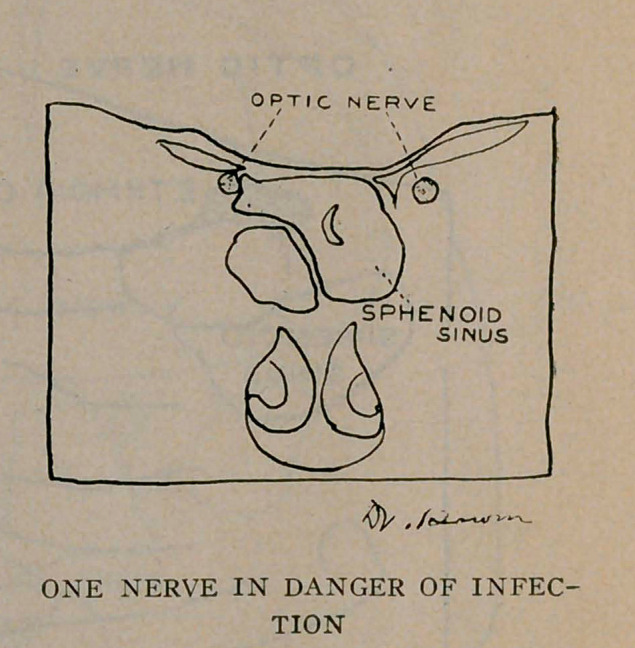


**Figure f5:**
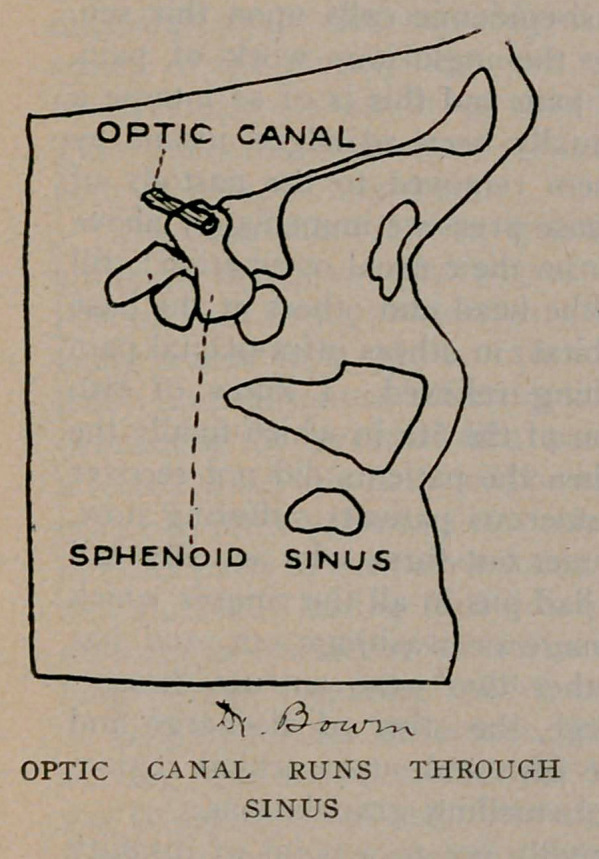


**Figure f6:**